# Fungal Keratitis in Northwestern Spain: Epidemiology, Risk Factors and Outcomes

**DOI:** 10.3390/jof10100689

**Published:** 2024-10-01

**Authors:** David Lamas-Francis, Daniel Navarro, Raquel Mansilla, Victoria de-Rojas, Claudio Moreno, Enrique Dios, Jesús Rigueiro, Dolores Álvarez, Paloma Crego, Teresa Rodríguez-Ares, Rosario Touriño

**Affiliations:** 1 Department of Ophthalmology, University Hospital of Santiago de Compostela, Ramón Baltar s/n, 15706 Santiago de Compostela, Spain; 2 Department of Microbiology, University Hospital of Santiago de Compostela, 15706 Santiago de Compostela, Spain; 3 Department of Ophthalmology, University Hospital of Vigo, 36312 Vigo, Spain; 4 Department of Ophthalmology, University Hospital of A Coruña, 15006 A Coruña, Spain; 5 Department of Ophthalmology, University Hospital of Ourense, 32005 Ourense, Spain; 6 Department of Ophthalmology, University Hospital of Pontevedra, 36161 Pontevedra, Spain; 7 Department of Ophthalmology, University Hospital Lucus Augusti, 27003 Lugo, Spain; 8 Department of Ophthalmology, University Hospital of Ferrol, 15401 Ferrol, Spain; 9 Department of Ophthalmology, Hospital Público da Mariña, 27880 Burela, Spain

**Keywords:** keratitis, cornea, fungi

## Abstract

Purpose: To review the clinical features, risk factors, microbiological profile, and treatment regimens of fungal keratitis in Galicia, a region in Northwestern Spain with temperate humid weather. Patients and methods: A retrospective case series was employed, including patients with fungal keratitis from nine hospitals within the region of Galicia, Spain, between 2010 and 2020. Data obtained from clinical records were analysed. Results: Out of 654 cases of infectious keratitis, 77 cases (9.9%) were identified as fungal keratitis. The median age of affected patients was 68.0 years, with a higher incidence in rural areas (62.3%). *Candida* spp. infections were the most frequent type (55.8%) and were associated with a higher median age than were the non-dermatophyte mould infections. The primary risk factors included steroid eyedrop use (29.9%), recent keratoplasty (18.2%), ocular trauma (19.5%), and contact with vegetable matter (11.7%). Most ulcers displayed stromal involvement, and 37.7% presented corneal thinning. The median duration of infection was longer in fungal than in bacterial keratitis, and surgical intervention was required in 48.1% of cases. Conclusions: Fungal keratitis, mainly involving *Candida* spp., accounted for 9.9% of microbial keratitis cases in Galicia, Spain, with significant risk factors being topical steroid use, ocular trauma, and contact with vegetable matter. Delayed diagnosis often resulted in poor outcomes, highlighting the need for early detection through awareness and new technologies to improve prognosis.

## 1. Introduction

Fungal keratitis is a potentially severe ocular infection that leads to a high incidence of preventable blindness and ocular evisceration [1]. The pathogenesis of fungal keratitis begins with disruption of the corneal epithelium, typically through trauma involving organic matter, such as plant material or soil, or through contact lens use [2]. Fungi use adhesins to facilitate their attachment to corneal epithelial cells and extracellular matrix proteins such as laminins, fibronectins, and collagens. Once attached, fungal spores germinate and produce hyphae within the corneal stroma. Fungi secrete enzymes like proteases and collagenases to invade and spread in corneal tissue, causing extensive damage. Fungal components are recognized by pattern recognition receptors (PRRs) like Toll-like receptors (TLRs) on corneal and immune cells, producing pro-inflammatory cytokines and IL-1ß that mediate neutrophil recruitment from the limbal blood vessels, which aid in killing fungal hyphae but also lead to significant corneal opacification [3,4].

Clinical symptoms and biomicroscopic features should prompt corneal scraping for culture to obtain a definitive diagnosis. These indications include ocular pain, foreign body sensation, blurred vision, photophobia, and increased tearing, as well as uneven fluffy margins, satellite lesions, endothelial plaques, and hypopyon [5]. Since bacterial keratitis is usually more common, and early clinical examination does not distinctly indicate fungal keratitis, many patients are often misdiagnosed and improperly treated. Corneal scraping for microbiological analysis, including microscopic examination and culture, is the standard method for diagnosis. While molecular methods offer faster diagnosis, with high sensitivity and specificity, they are not yet routine in clinical practice [6]. Non-invasive imaging techniques include in vivo confocal microscopy, which allows for direct observation of fungal hyphae in the cornea, and optical coherence tomography (OCT), among others.

Management involves a topical–systemic-targeted strategy, where the initial treatment should be topical natamycin eyedrops. Polyenes, such as natamycin or amphotericin B, target fungal cells by binding to ergosterol in the cell membrane, forming pores that increase membrane permeability and lead to cell death. The Mycotic Ulcer Treatment Trial found that natamycin was more effective than voriconazole, especially in cases caused by *Fusarium* spp. [7]. Azoles, including voriconazole, disrupt ergosterol synthesis, impair cell membrane function, and are generally reserved for rare species or in cases of resistance to polyenes [8,9]. Topical antifungal treatments are limited by poor corneal penetration and fungistatic effects, which reduce their therapeutic success. Systemic medication (ketoconazole or voriconazole) is typically prescribed for larger and deeper ulcers, or when the infection spreads to intra- or extraocular tissues. Additional techniques, such as intrastromal and/or intracameral injections of voriconazole, may be considered [10]. Delays in diagnosis, prior use of steroid eye drops, a large variety of pathogens, and limited antifungal agents contribute to poor outcomes in fungal keratitis, with a large percentage of cases requiring surgical intervention, including ocular evisceration [11]. Other techniques, such as amniotic membrane transplantation and corneal-crosslinking, have been explored; however, their efficacy is not fully established. Therapeutic penetrating keratoplasty is effective in restoring corneal integrity and eradicating infections, especially when combined with intravitreal antimicrobials. Surgical interventions are more frequently required in fungal than in bacterial keratitis, underscoring its poor response to medical treatment. Prophylactic antifungal treatment is recommended in high-risk cases [12].

More than 70 species of yeasts and filamentous fungi can cause keratitis. Fungal keratitis is more common in warm and humid climate regions such as India and southern China, especially cases caused by non-dermatophyte mould species such as *Fusarium* spp. and *Aspergillus* spp., with fungal isolates representing up to 60% of the large series of microbial keratitis [13,14]. On the other hand, yeasts such as *Candida* spp. are more common in developed countries and regions with colder climates, where steroid use and contact lens use are more important risk factors [15]. The frequency of fungal keratitis in the European series ranges between 3 and 9% of all cases of microbial keratitis [16,17]. Other risk factors include fungal skin disease, lacrimal duct obstruction, ocular surface disease, herpetic keratitis, ocular surgery, eyelid malposition, etc.

In this multicentric study, we present the epidemiology, risk factors, management, and outcomes of a series of fungal keratitis cases diagnosed between 2010 and 2020 in the region of Galicia, a region in Northwestern Spain with a temperate and humid climate. Understanding the local microbiological profile of fungal keratitis is important in order to guide appropriate treatment.

## 2. Patients and Methods

This retrospective study included all patients diagnosed with infective fungal keratitis between 2010 and 2020 in nine public hospitals in Galicia, Spain. The participating centres included seven tertiary hospitals (university hospitals of Santiago de Compostela, A Coruña, Vigo, Pontevedra, Lugo, Ourense, and Ferrol, Spain) and two public secondary hospitals (Burela and Monforte de Lemos, Spain). The electronic database of each microbiology department was searched to select patients who presented with a corneal infiltrate and had a positive culture for a fungal isolate after corneal scraping. Other causes of microbial keratitis and patients with insufficient data were excluded.

Electronic medical records of a total of 654 infectious keratitis cases were reviewed, and the following variables were extracted: demographic features, risk factors, and clinical examination data, including distance best-corrected visual acuity (DBCVA), a description of the epithelial defect, and the infiltrate and anterior chamber reaction. The microbiological profile of the fungal isolates and the treatment used in each case were noted, including the need for a surgical procedure. The standard protocol involved inoculating corneal tissue from corneal scraping onto chocolate agar and Sabouraud media. In cases where a protozoan infection was suspected, a non-nutrient agar plate seeded with *E. coli* was also used. Cases with a positive fungal culture were considered infectious if there was also a clinical correlation, such as the presence of characteristic biomicroscopic signs of fungal keratitis and a favourable response to antifungal treatment. Positive cultures that did not meet these criteria were excluded from the study, as they were considered potential contaminants.

This study follows the principles outlined in the Declaration of Helsinki and received approval from the Santiago-Lugo Research Ethics Committee (2019/148). Since the data were collected retrospectively from electronic medical records based on routine clinical practice and did not impact patient care or outcomes, informed consent was waived.

Demographic data, clinical characteristics, and microbiological isolates were summarized using descriptive statistics, including the median and interquartile range for non-normally distributed variables, as well as frequencies and percentages for categorical variables. The *t*-test was used for normally distributed variables, the Mann–Whitney test for non-normally distributed variables, and the Chi-square test for categorical variables. The Yates’ correction was used when the expected frequency was less than five. The Chi-square test for goodness of fit was conducted to determine whether the distribution of samples was significantly different across seasons. A paired *t*-test was used to assess the significance of the differences in DBCVA. A multivariate logistic regression model was constructed to determine the factors associated with fungal keratitis relative to those associated with bacterial keratitis. Statistical significance was assessed at the 0.05 level. The statistical analysis was conducted using SPSS v23.0.

## 3. Results

**Figure 1:**
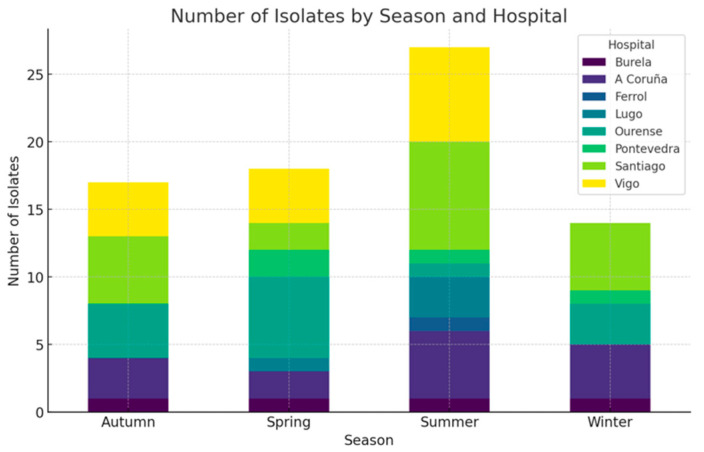
Seasonal distribution of fungal isolates in each centre.

A total of 77 cases of fungal keratitis were identified, accounting for 9.9% of the 654 infective keratitis cases in our study series. A total of 38 patients were male (49.4%), and 39 were female (50.6%). The median age at diagnosis was 68.0 years (IQR 56.0–80.0). Most of the cases, 48 (62.3%), were from rural areas, while 29 cases (37.7%) were from urban areas. There were no differences in the incidence of yeasts (*n* = 43) or non-dermatophyte moulds (*n* = 34) associated with rural or urban residence (Chi-square, *p* = 0.790). Patients with *Candida* spp. infections were older (median age, 72.0 years) than those with non-dermatophyte mould infections (median age, 62.0 years) (Mann–Whitney U test, *p* = 0.024). The infections affected the right eye in 45 cases (58.4%) and the left eye in 32 cases (41.6%). The seasonal distribution of cases showed that 18 cases (23.4%) occurred in winter, 19 cases (24.7%) in spring, 23 cases (29.9%) in summer, and 17 cases (22.0%) in autumn (Figure 1). Although the cases were identified more frequently in summer, the number was not statistically significant (Chi-square goodness of fit, *p* = 0.782).

The main patients characteristics which are typically considered risk factors for developing keratitis were recorded and are shown in Table 1. The multivariate model showed that contact with vegetable matter (*p* = 0.032) and topical steroids (*p* = 0.011) were factors associated with fungal keratitis rather than with bacterial keratitis (Table 2). There were no differences in the number of infections caused by yeasts or non-dermatophyte moulds (Chi-square, *p* = 0.830) associated with the use of contact lenses. 

The median DBCVA was 1.0 logMAR (IQR 0.1–2.4) before the infection, decreased to 2.1 logMAR (IQR 0.7–2.4) at the time of diagnosis, and improved to 1.3 logMAR (IQR 0.4–2.4) at the last follow-up available. While the median value of the final DBCVA was slightly higher, the difference relative to the baseline DBCVA was not statistically significant (paired *t*-test, *p* = 0.179). This analysis did not consider eyes which were eviscerated. The final DBCVA was not significantly different between infections resulting from *Candida* spp. and those caused by other fungal infections (*t*-test, *p* = 0.403). The median duration of the infectious process (from diagnosis to epithelial defect closure) was 48 days (IQR 25.2–144.2 days), while the median time from diagnosis to corneal scraping was 5 days (IQR 0–15 days). Corneal scraping was performed significantly later in cases of fungal than in cases of bacterial keratitis (0 days, IQR 0–2) (*t*-test, *p* < 0.001). Compared to bacterial keratitis in our series (median duration of 22 days, and 0 days from diagnosis to corneal scraping), fungal keratitis displayed a significantly longer infection duration and time from diagnosis to corneal scraping (Mann–Whitney test, *p* < 0.001).

The biomicroscopic findings are shown in Table 3. Most ulcers presented a single infiltrate with stromal involvement and affected the central 2 mm of the cornea. The frequency of anterior chamber reaction and significant corneal thinning was relatively high.

The microbiological profile of our series is provided in Table 4. Yeasts were the most common fungi observed in our study, accounting for 55.8% of all cases. The remaining cases were identified as non-dermatophyte moulds, with *Fusarium* spp. being the most prevalent. A total of 30 cases (39.0%) of keratitis were polymicrobial. Apart from the fungus isolated, a Gram-positive bacterium was found in 17 cases (15.6), a Gram-negative bacterium in 5 cases (4.6%), and both Gram-positive and Gram-negative bacteria in two cases (1.8%). A combination of fungi was reported in three cases (2.8%). The antifungal susceptibility was not available for most cases, except for *Candida* spp., with only one isolate reported as resistant to voriconazole (3.8%) and the others being susceptible to the antifungals tested (amphotericin B, fluconazole, itraconazole, caspofungin, and micafungin). The *Candida* spp. isolate resistant to voriconazole was collected from a 62-year-old male with a recent keratoplasty, who eventually responded to topical treatment with a combination of antifungals.

Among the patients, 11 (14.3%) were hospitalized during the course of the infection. Initial empirical treatment strategies included fortified antibiotic eyedrops in 36 patients (46.8%), and commercial antibiotic eyedrops were used in 34 patients (44.2%). Topical antifungal treatment was initiated at the time of diagnosis due to strong clinical suspicion or the detection of hyphae in the Gram staining. The initial treatment regimens and the administration of systemic medication are shown in Table 5. Surgical intervention was required in 38 patients (48.1%). Both the need for surgical intervention in general and evisceration specifically were more frequent in cases of fungal compared to bacterial keratitis in our series (Chi-square, *p* = 0.002 and *p* < 0.001). Some patients underwent more than one procedure. Risk factors for evisceration inlcuded previous herpetic keratitis (*p* = 0.012) and previous ocular surgery (*p* = 0.008), although previous keratoplasty and cataract surgery were protective factors.

## 4. Discussion

This study shows the epidemiology and outcomes of fungal keratitis in the Northwestern region of Galicia (Spain) between 2010 and 2020. Fungal keratitis represented 9.9% of all the cases of microbial keratitis during this period, which is in line with the results presented in similar recent publications in occidental regions. Galicia is a coastal region with 2.7 million inhabitants, with an aging population living predominantly in rural areas, where agricultural activities are common. The weather is temperate, with abundant precipitation and high relative humidity.

Cases of fungal keratitis peaked in summer months, with a reduced incidence in colder months. Seasonal fluctuations may be related to certain activities that are more frequent during these months, such as the increased use of contact lenses, ocular trauma, and agricultural activities. Ting et al. reported a comparable trend in a similar climate (Nottingham, UK) [16], while Lin et al. suggest a connection between the harvesting season and contact with dust in the wind during summer, with an increase in the incidence of fungal infections in South India, where the temperatures are persistently hot [18].

*Candida* spp. was the most common fungal isolate in our series, as was also the case in other series with similar socioeconomic and climatic settings [19,20,21]. Contact lens wear, recent ocular surgery, and steroid eyedrop use were present in approximately one-third of patients in our study. Contact lens use and poor contact lens hygiene are believed to increase the adherence of microorganisms to the cornea, which increases the risk of infection, especially when an epithelial defect is present. Some patients with bullous keratopathy or other chronic conditions (severe dry eye disease, neurotrophic ulcers) require the use of therapeutic contact lenses, as well as short cycles of topical steroids, which can also lead to infection, especially in older or immunosuppressed patients. We found that patients with infections caused by *Candida* spp. were older than those with keratitis caused by non-dermatophyte moulds. Filamentous fungi, including non-dermatophyte moulds, are more commonly isolated in contact lens wearers [19], while local immunosuppression, caused by ocular surface disease, recent surgery, and topical steroids, is linked to keratitis caused by *Candida* spp. [22]. Prolonged use of steroids before the diagnosis is common, as fungal keratitis is usually misdiagnosed. Steroids are often incorporated into treatment regimens after a poor clinical response to antibiotic eyedrops. Steroids have been shown to contribute to increased aggressivity of the fungi and a limited immunological reaction, resulting in worse outcomes in animal models and cases of fungal keratitis [23].

Variable rates of azole resistance have been observed in clinical isolates of *Candida* spp., depending on the subspecies and specific antifungal used [24]. Up to 3% of *Candida* spp. isolates are resistant to voriconazole and often show multidrug resistance or reduced susceptibility to other azoles [25]. The molecular mechanisms behind antifungal resistance include altered drug affinity, where mutations or overexpression in target genes, such as *ERG11* for azoles, reduce drug efficacy; reduced intracellular drug levels caused by the increased activity of efflux pumps; and the formation of biofilms [26]. Fungi may also survive drug-induced stress through the inhibition of proteins such as Hsp90, and genomic plasticity helps fungi rapidly adapt and develop resistance to antifungal agents [27].

Contact with vegetable matter and the use of steroid eyedrops prior to the infection were significantly more common in fungal keratitis than in the overall group. Fungi and plants interact in different ways, including through symbiotic and pathogenic associations. Species of *Fusarium* spp., *Aspergillus* spp., *Paecilomyces* spp., and *Alternaria* spp., among others, are known to cause significant disease in plants and can come in contact with the ocular surface following trauma with vegetable matter or can be carried by the wind [28]. Non-dermatophyte moulds, other than *Aspergillus* spp. and *Fusarium* spp., are generally rare causative agents of fungal keratitis, although we isolated them at a relatively high frequency in our study. Over one-third of the patients in this series had experienced recent ocular surgery, with 18% having undergone corneal transplantation. Ocular surgery can be a significant risk factor for corneal infection because it often compromises the epithelial barriers, particularly in corneal procedures that involve sutures and prolonged use of topical steroids. Penetrating and deep-anterior lamellar keratoplasties are more commonly caused by surface commensals (Gram-positive bacteria), whereas endothelial keratoplasties are usually caused by *Candida* spp. [29]. A total of 20% of the patients in our series had experienced recent ocular trauma, and 12% reported contact with vegetable matter before the infection. This was significantly lower than the reported frequency of up to 92% in other studies, where plant pathogen fungi were more commonly isolated [30,31] in warmer and humid regions with more agricultural activities. A summary of recent case series of fungal keratitis, including the risk factors present and the most commonly isolated species, is presented in Table 6. Understanding the risk factors can provide valuable insights into the underlying causative organisms, potentially guiding the selection of antifungal treatment, especially in cases of negative cultures.

The initial treatment in most cases included the use of topical antibiotics. This is explained by the initial delay in the definitive diagnosis, with milder cases being prescribed antibiotics, and antifungals being initiated empirically only in severe cases with a high suspicion of a fungal origin. The fact that a culture was taken 5 days (median) after the initial visit (compared to a median of 0 days in bacterial keratitis) explains this delay in diagnosis. This pattern has been described in most studies, where fungal keratitis is not as common as bacterial keratitis [19,32]. A longer period without appropriate treatment, especially in the older population, probably exacerbated cases, leading to larger and deeper infiltrates, with a consequently longer healing time. This has also been described for cases with prolonged use of steroids before the infection, with worse disease progression and treatment outcomes [38]. The use of steroids in the treatment of fungal keratitis is generally contraindicated [23].

Almost half of the patients in our series required surgical intervention. The need for both surgery and evisceration were more frequent in cases of fungal than in bacterial keratitis, with 18% of eyes being eviscerated. This is an indicator of the poor prognosis of fungal keratitis. Prajna et al. showed that the presence of hypopyon, as well as infiltrates involving the inner third of the corneal depth, were predictors for perforation and the eventual need for therapeutic penetrating keratoplasty [39]. The emergence of rare cases of fungal keratitis with limited therapeutic options can also require evisceration [40]. Confocal microscopy, as well as new technologies, such as deep learning models based on slit-lamp photography and/or OCT, may be able to accelerate diagnostic times and provide valuable support in the early identification of potential fungal keratitis, enabling timely management [41].

This study presents several limitations. The retrospective design inherently includes the risk of missing or incomplete data in electronic medical records. Some risk factors or clinical features may have been omitted, if not specifically documented in the clinical history, potentially leading to an erroneous importance given to each variable. In vitro antifungal susceptibility testing was not always performed and may not be representative of the clinical response when available, as specific cut-off points for ocular administration have not been established.

In summary, fungal keratitis represented 9.9% of all cases of microbial keratitis in Galicia (Spain) during the 11-year study period. *Candida* spp. was the most commonly isolated fungus. The most important risk factors for fungal infection were contact with vegetable matter and the use of topical steroids. The significant delay in diagnosis led to relatively poor outcomes, with a significant rate of eviscerated eyes. Understanding the risk factors and detecting suggestive clinical signs through new technologies may help identify cases earlier, thereby improving the prognosis.

## Figures and Tables

**Table 1:** Clinical characteristics of patients with fungal keratitis and other forms of microbial keratitis (bacterial and amoebal keratitis).

Associated Factor	Fungi	%	Bacteria and Amoebae	%	*p*-Value
Diabetes	11	14.3	94	16.3	0.796
Systemic immunosuppression	5	6.5	24	4.2	0.926
Systemic steroid use	2	2.6	11	1.9	0.980
Recent ocular surgery	28	36.4	190	32.9	0.705
Recent keratoplasty	14	18.2	62	10.7	**0.043**
Contact lens use	21	27.3	137	23.7	0.563
Recent ocular trauma	15	19.5	60	10.4	**0.033**
Contact with vegetable matter	9	11.7	23	4.0	**0.002**
Foreign corneal body	3	3.9	18	3.1	0.931
Steroid eyedrop use	23	29.9	100	17.3	**0.004**
Glaucoma	19	24.7	140	24.3	0.721
Blepharitis	12	15.6	121	21.0	0.350
Eyelid disorders	8	10.4	76	13.2	0.375
Previous keratitis	20	26.0	175	30.3	0.598

**Table 2:** Multivariate regression model assessing risk factors for fungal keratitis in comparison with bacterial and amoebal keratitis.

	Coefficient	Standard Error	Wald	Degrees of Freedom	Sig.	Odds Ratio (OR)
Trauma	0.187	0.449	0.174	1	0.677	1.206
Recent keratoplasty	0.553	0.345	2.569	1	0.109	1.738
Previous topical steroids	0.728	0.289	6.325	1	**0.012**	2.071
Vegetable matter	1.247	0.565	4.872	1	**0.027**	3.478
Constant	−2.433	0.168	208.798	1	0.000	0.088

**Table 3:** Summary of main biomicroscopic features in patients with fungal keratitis.

Biomicroscopic Feature		N	%
Epithelial defect	Small (<3 mm)	36	46.8
	Large (>3 mm)	32	41.6
Infiltrate number	1	55	71.4
	2	6	7.8
	>2	9	11.7
Infiltrate depth	Superficial	10	13.0
	Stromal	49	63.6
	Endothelial plaque	8	10.4
Infiltrate localisation	Central 2 mm	32	41.6
	Paracentral	25	32.5
	Peripheral 2 mm	7	8.3
Corneal thinning	Thinning	29	37.7
	Perforation	19	24.7
Anterior chamber reaction	Tyndall	15	19.5
	Hypopyon	22	28.6
Endophthalmitis		6	7.8

**Table 4:** Frequency of species of fungi isolated from corneal scraping cultures.

Isolate	No. of Isolates	%
**Yeasts**		
*Candida* spp.	43	55.8
**Non-dermatophyte moulds**		
*Fusarium* spp.	13	16.9
*Aspergillus* spp.	6	7.8
*Paecilomyces* spp.	4	5.2
*Alternaria* spp.	3	3.9
*Acremonium* spp.	3	3.9
*Stemphylium* spp.	1	1.3
*Albifimbria* spp.	1	1.3
*Scedosporium* spp.	1	1.3
*Curvularia* spp.	1	1.3
Not identified	1	1.3

**Table 5:** Summary of treatment regimens in cases of fungal keratitis.

Treatment Regimen		N	%
Initial topical treatment	Fortified antibiotics	36	46.8
	Commercial antibiotics	34	44.2
	Voriconazole	3	3.9
	Voriconazole + amphotericin B	2	2.6
	Voriconazole + amphotericin B + natamycin	1	1.3
	Amphotericin B	1	1.3
Topical steroids		37	48.0
37	Antifungals	23	29.9
	Steroids	6	7.8
Surgery	Penetrating keratoplasty	17	22.1
	Deep anterior lamellar keratoplasty	2	2.6
	Amniotic membrane transplantation	6	7.8
	Acrylic glue application	9	11.7
	Stromal antifungal injection	3	3.9
	Evisceration	14	18.2

**Table 6:** Summary of risk factors and most common fungal species noted in recent publications.

Author	Region	Study Period	N	Contact Lens Use	Trauma	Ocular Surface Disease	Ocular Surgery	Topical Steroids	Diabetes	Most Common Fungal Isolate
America										
Keay et al. [20]	USA	2001–2007	733	37%	25%	29%				*Candida* spp.
Atta et al. [32]	Pittsburgh (USA)	2015–2021	28	68%	43%	32%	43%	32%		*Aspergillus* spp. and *Fusarium* spp.
Trinh et al. [21]	Toronto (Canada)	2020–2021	46	39%	9%			70%		*Candida* spp.
Southeast Asia										
Ghosh et al. [33]	Chandigarh (India)	2005–2011	393		32%	6%		1%		*Aspergillus* spp.
Bharathi et al. [31]	Tamil Nadu (India)	1999–2002	1095		92%	7%		1%	15%	*Fusarium* spp.
Soleimani et al. [34]	Tehran (Iran)	2019–2021	86	6%	49%		12%	3%	7%	*Fusarium* spp.
West Pacific										
Dan et al. [30]	Shandong (China)	2010–2016	851		55%	3%				*Fusarium* spp.
Kim et al. [35]	Darwin (Australia)	2014–2022	31	45%	32%					*Curvularia* spp.
Africa										
Cheikhrouhou et al. [36]	Sfat (Tunisia)	1995–2012	60	3%	50%	10%	10%	18%	5%	*Fusarium* spp.
Sadik et al. [37]	Mansoura (Egypt)	2018	171	4%	32%	25%	23%	2%	7%	*Aspergillus* spp.
Europe										
Ting et al. [19]	Nottingham (UK)	2011–2020	51	45%	9%	76%	27%	21%		*Candida* spp.
Lamas-Francis et al. (present study)	Galicia (Spain)	2010–2020	77	27%	19%		36%	30%	14%	*Candida* spp.

## Data Availability

The original contributions presented in the study are included in the article, further inquiries can be directed to the corresponding authors.
